# Effects of Single or Multiple Sessions of Whole Body Vibration in Stroke: Is There Any Evidence to Support the Clinical Use in Rehabilitation?

**DOI:** 10.1155/2018/8491859

**Published:** 2018-07-30

**Authors:** Cosimo Costantino, Federica Petraglia, Laura Luigia Sabetta, Riccardo Giumelli

**Affiliations:** ^1^Department of Medicine and Surgery, University of Parma, Italy; ^2^Physical Medicine and Rehabilitation Residency Program, University of Parma, Italy; ^3^Pre-Med Student, University of Parma, Italy

## Abstract

**Background and Purpose:**

Recently new technologies and new techniques, such as Whole Body Vibration (WBV), have been introduced by the health and fitness industry to pursue therapeutic or physical performance goals. The aim of this systematic review is to investigate the effectiveness of single or multiple WBV sessions alone or in association with traditional rehabilitation, compared to traditional rehabilitation therapy or with sham therapy in poststroke patients.

**Methods:**

Randomized Control Trials and controlled clinical trials written in English between January 1st, 2003, and December 31st, 2017, were selected from PubMed, Cochrane-Central-Register-of-Controlled-Trials, and Physiotherapy-Evidence-Database (PEDro). The single WBV session and multiple sessions' effects were assessed. Study characteristics, study population, intervention protocols, effects of WBV sessions, and adverse events were investigated with a descriptive analysis.

**Results:**

The search reported 365 articles and after screening and removal of duplicates, 11 manuscripts with PEDro score≥6/10 were selected (391 poststroke patients). Study characteristics, study population, intervention protocols (frequencies, amplitude of vibration, and peak acceleration), effects of a single or multiple WBV sessions, and adverse events were analyzed. They have been investigated with particular attention to bone turnover, structure and muscle functions, spasticity, postural control and risk of falls, functional mobility, somatosensory threshold, and activity and participation. Comparing WBV group with control group no significant benefits emerged.

**Discussion:**

This systematic review included studies involving participants with non homogeneous characteristics, just considering the incorporation of studies on individuals with chronic and postacute stroke. Despite these limits, WBV treatment has no significant risks for patients and shows interesting effects of WBV treatment in Structure and muscle functions, Spasticity and Postural control.

**Conclusions:**

Even though treatment with WBV appears safe and feasible, there is insufficient evidence to support its clinical use in poststroke rehabilitation at this point. More studies assessing other functional tests and with more specific treatment protocols are needed.

## 1. Introduction

Recently new technologies and new techniques, such as Whole Body Vibration (WBV), have been introduced by the health and fitness industry to pursue therapeutic or physical performance goals. Basic neurophysiological studies have shown that vibration can alter sensory and motor function by mostly activating the primary spindle endings, although secondary spindle endings, such as Golgi tendon organs, Pacinian, and Meissner corpuscles can also be activated [1]. Several types of Whole Body Vibration platforms can be found in literature [2–4].

Currently, there are three commercial typologies of vibration platforms. The first one, Galileo®, has a teeterboard that produces asynchronous sinusoidal side-alternating vertical vibrations.

The second type of commercial machines (Bodypulse®, Power Plate®, Soloflex®, Nemes®, Vibra Pro®, Vibra Fit®, Fitvibe®, PneuVibe®, and VibroGym®) produces vertical synchronous vibrations. The third type, called Extream 1000 AMH International Inc., Korea, is a slipping platform that produces horizontal vibrations [5].

Key descriptors of vibration devices include the frequency (number of complete movement cycles per second, measured in hertz), the amplitude (displacement of oscillatory motion, measured in mm), the acceleration (measured in m/s^2^ or g), and the duration (exposure time) of the vibration exposure [6]. The intensity of vibration is determined by varying both frequency and amplitude; accordingly it may be possible to get a training program tailored to the needs of the person, or to adapt it to different goals.

The vibration devices can differ with frequency ranges from 0 to 60 Hz, amplitudes from 0 to 12 millimeters, and peak acceleration from 0 to 20,1 g. In a typical session, the user stands on the device doing static or dynamic exercises while the platform produces sinusoidal oscillations. In most cases, the vibration session consists of several bouts of vibration exposure (each lasting from less than a minute to several minutes) separated by rest periods.

The growing interest in vibrations started from animal research in the 1990s and early 2000s when a correlation between vibration and bone deposition was reported [7, 8].

Other studies demonstrated that WBV training causes a continuous proprioceptive stimulation which increases neuromuscular receptivity [9]. Many studies have highlighted the possibility of WBV training to improve sport performance, increasing range of motion, and to be a beneficial supplementary training technique in strength programs for athletes [10–16].

Others studies have explored WBV applications in different clinical frameworks such as Osteoarthritis [17], Cognitive Function [18, 19], Postmenopausal Women [20, 21], Spinal Cord Injury [22], Rheumatoid arthritis [23], Multiple Sclerosis [24], Parkinson's disease [25], Down Syndrome [26], Metabolic Syndrome [27], Osteoporosis [28], Chronic Obstructive Pulmonary Disease [29], and other medical conditions [30].

The aim of this systematic review is to investigate the effectiveness of single or multiple WBV sessions, alone or in association with traditional rehabilitation, compared to traditional rehabilitation therapy or with sham therapy in patients with a stroke.

## 2. Methods

### 2.1. Study Design and Eligibility Criteria

This systematic review was conducted and reported in accordance with Preferred Reporting Items for Systematic Reviews and Meta-Analyses (PRISMA statement). We have used PICO method (Patients/Population, Intervention, Comparison, Outcomes) [31] and a qualitative analysis focused on the differences between the selected studies. We followed PICO variables: persons with stroke (P); WBV training (I); comparison between WBV therapy and the same exercises performed without WBV, comparison between WBV therapy and other physical activities or sham therapy (C); outcomes measuring body functions and structures, activities, and participation (O) as reported in International Classification of Functioning, Disability and Health (ICF) Stroke Brief [32]. We investigated the effects of WBV therapy on patients with ischemic or hemorrhagic stroke. Only Randomized Control Trials (RCT) and controlled clinical trials written in English were selected. The single WBV session and multiple sessions effects were assessed.

We excluded studies on animals; not about stroke; based on focal vibration treatments; with a PEDro score<6 [5, 33, 34], or where the full-text was not available in our institutional University Library System.

### 2.2. Data Sources and Searches

We selected all papers published from January 1st, 2003, until December 31st, 2017, in the following electronic databases: PubMed [35], Cochrane-Central-Register-of-Controlled-Trials [36], and Physiotherapy-Evidence-Database (PEDro) [37].

The search query, based on the PICO strategy, included both ischemic and hemorrhagic stroke. The string used for PubMed was launched in the first week of January 2018 and contained at least one of these terms: “Nervous System Disease”, “Stroke”, Whole Body Vibration”, “Vibration”, “vibration platform”, “sham therapy”, “rehabilitation therapy”, “gait”, “balance”, “muscle performance”, “spasticity”, “bone turnover”, “postural control”, and “muscle strength”.

Those keywords were used in several combinations with Boolean operators (AND/OR) and modified for other databases.

### 2.3. Levels of Evidence

Study quality was assessed according to the guidelines of the Oxford Centre for Evidence- Based Medicine [38]; we have assigned a level of evidence 2 to all the studies included in this systematic review. To assess the methodological quality of the selected studies we used the PEDro scale [39], considering only high quality studies (score≥6). The results of methodological quality assessment are displayed in Table 1.

### 2.4. Data Extraction

Articles were initially screened by title and abstract. Articles unclear from their title or abstract were reviewed according the selection criteria through full-text. Three authors (F. P., L. S., and R. G.) independently extracted data from the studies that met the inclusion criteria and they were blinded to each other's review. In case of disagreement, a fourth opinion (C. C.) could have been requested. Conference abstracts were evaluated but deemed not suitable because of the limited body of data related to the study.

### 2.5. Data Synthesis and Analysis

We performed a descriptive analysis of the measures of WBV effects on each outcome selected. The heterogeneity of outcomes, participants, and intervention protocols made it impossible to draw up a meta-analysis. In the articles with significant outcomes we calculated the changes among the groups using the values of SES (Standardized-Effect-Size) concerned. The calculation was performed using the average values and standard deviations. The effect size was considered, according to Hedges [51], small (for values of SES = 0.2), medium (SES = 0.5), and large (SES = 0.8).

## 3. Results

### 3.1. Study Selection

**Figure 1:**
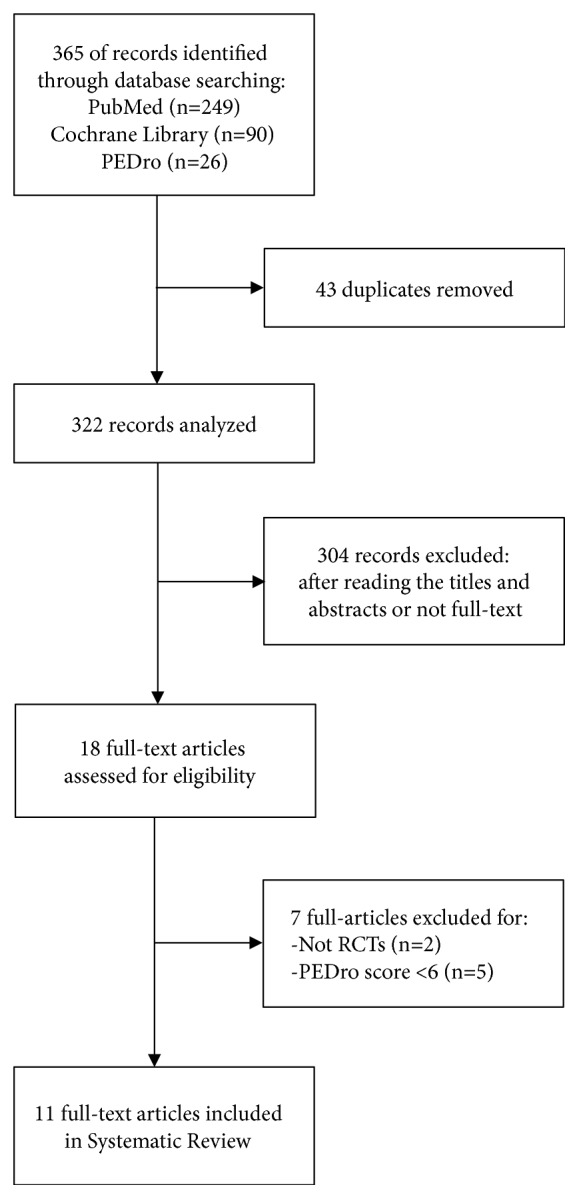
Flow diagram: phases of the systematic review.

Figure 1 describes each step of our database research. Our initial search on PubMed produced 249 records, plus 90 records from Cochrane Library and 26 from PEDro Database. After removing 43 duplicates, an assessment was performed on headlines, abstracts, and full texts, which resulted in the removal of 304 records that left 18 eligible articles. Among the remaining 18 eligible articles 2 were not RCTs and 5 had PEDro scores<6/10. Therefore in this systematic review were included 11 articles [40–50] (10 studies). The two reports by Lau et al. [41] and Pang et al. [42] are based on identical data.

### 3.2. Study Characteristics

To assess the methodological quality of the selected studies we used the PEDro scale [39], considering only high quality studies (score≥6) (Table 1). Only Brogårdh et al. [43] matched subjects, therapist, and assessor blinding (9/10 score).

### 3.3. Study Population

Patients were recruited from a Rehabilitation Center [40, 43–45, 47, 49, 50] (7 studies), an association that included people with stroke [46] (1 study); a local self-help group for people with stroke [41, 42] (1 study); or not specified [48] (1 study). Eight clinical trials involved patients with chronic stroke (onset≥6 months) [41–48, 50] and 2 with postacute stroke (a few days after stroke) [40, 49]. Furthermore, 391 poststroke patients were involved, 129 women and 262 men (mean age 59.74 years). Only Tihanyi et al. [40] has provided a single value of mean age (58.2± 9.4) common to both groups; other studies presented differences or substantial gaps [50] in age between groups. Not all studies clarified the stroke nature (ischemic/hemorrhagic) or location (left/right). Participants characteristics are summarized in Table 2.

### 3.4. Intervention Protocol for WBV Group

There are significant differences in the WBV protocols (Table 3): frequencies ranged from 5 to 40Hz, amplitude of vibrations from 0.44 to 5.8mm, and peak acceleration of the vibrations from 0.2 to 16.1g (gravitational constant). Liao et al. 2016 [46] investigated the effects of vibration intensity in poststroke patients. Two groups performed exercises on the same vibrating platform, with the same amplitude but with different frequencies and acceleration (respectively, 20 and 30Hz and 1.61 and 3.62g).

Six studies used a vertical synchronous vibration [40, 41, 43, 44, 46, 50] and four studies used an asynchronous vertical sinusoidal vibration transmitted alternately to the left and right side of the body [45, 47–49].

In all studies the vibrations were delivered in bouts (from 1 to 17 discharges, for a duration of 15 to 180 seconds each) with short rest periods. Two studies [40, 44] evaluated the immediate effects of a single WBV session and 8 trials [41–43, 45–50] examined the effects of multiple WBV sessions (duration 4-12 weeks, frequency 1-5 sessions per week).

Five studies [40, 43–45, 49] have provided only static exercises on WBV. The most common static exercise used was the semisquat with knee flexion at 30° and 60° while standing on the vibratory platform. Five other studies [41, 42, 46–48, 50] provided a set of static and dynamic exercises. In Marín et al. [45] the participants performed the exercises with WBV in addition to the daily conventional rehabilitation therapy. In Choi W et al. [48] participants performed the exercises with WBV combined with Treadmill Training.

In the Lau et al. [41] and Pang et al. [42] papers, participants completed 1.5 minutes of warm- up exercises in a sitting posture. Sessions in Choi W et al. [48] were preceded by 15 minutes of gentle stretching, while sessions were preceded by 10 minutes of warm-up and followed by 10 minutes of cool-down exercises in the Liao et al. [46] paper (Table 3).

### 3.5. Intervention Protocol for Control Group

In 8 studies the control group performed the same exercises, standing on the same platform, but without vibration [40–42, 44–48] or with sham vibration [43]. In 2 studies [49, 50] the control group performed conventional rehabilitation exercises with music or maintained habitual physical activity (Table 3).

### 3.6. Effects of a Single WBV Session

Tihanyi et al. 2007 [40] and Chan et al. 2012 [44] (46 participants) investigated the immediate effects of a single WBV session. In Table 3 are summarized the outcome measures including significant findings about lower limb muscle strength, spasticity, postural control, and functional mobility.

### 3.7. Effects of Multiple WBV Sessions

Eight studies (345 participants) investigated the effects of multiple WBV sessions, with a treatment duration of 4-12 weeks [41–43, 45–50] (Table 3). The significant findings for comparisons between WBV therapy and the same exercises performed without WBV included bone turnover, lower limb muscle strength/motor functions, muscle thickness, spasticity, postural control, falls, functional mobility, daily activities, and Stroke-Impact-Scale. The significant findings for comparisons between WBV therapy and other physical activities or sham therapy indicate muscle strength/motor functions, spasticity, postural control, sensory threshold, functional mobility, and daily activities.

### 3.8. Events during WBV Sessions

A total of 211 participants were exposed to WBV. Six trials [41–43, 45, 46, 49, 50] reported slight to mild side effects, generally declining after the first therapeutic sessions. In Lau et al. [41], 5 of the 41 participants in the WBV group reported adverse symptoms potentially related to vibration: knee pain, fatigue, and dizziness. Brogårdh et al. [43] reported that 15 of the 31 participants, in both groups, reported a transient and mild muscle soreness or muscle fatigue.

Tankisheva et al. [50] reported that some participants felt a tingling in the legs. Liao et al. [46] reported a participant with moderate knee pain after low-intensity WBV, 3 participants with fatigue after low-intensity WBV, and 2 after High-Intensity WBV. Two studies [45, 49] have no side effects in all participants (38 persons) in the WBV group. In 3 studies [40, 44, 47] it is not clear whether any adverse events occurred.

## 4. Discussion

Our literature shows that WBV treatment presents no significant risks for patients, but in this review we cannot state an objective benefit in poststroke patients according to ICF (e.g., bone turnover, motor functions, balance, mobility, somatosensory threshold, risk of falls, and activities of daily life and participation).

### 4.1. Bone Turnover

Literature shows an accelerated loss of bone mass in the paretic side [52], a high level of bone resorption, and a low level of markers of bone formation in poststroke patients [53].

In our review, Pang et al. [42] measured, with no significant results, biochemical markers of bone turnover (C-telopeptide of type I collagen cross links and bone-specific alkaline phosphatase). Since the current literature may present beneficial results of WBV for bone mineral density, further studies are necessary to investigate WBV effects to the bone of poststroke patients.

### 4.2. Structure and Muscle Functions

Five trials [41–43, 45, 46] did not show significant results. Tihanyi et al. [40] reported a variable muscle strength after a single WBV session: increase of maximum isometric knee extension torque (SES=0.50); increase of maximum eccentric knee extension torque (SES=0.46) on the paretic side; decrease of coactivation quotient of Biceps Femoral Muscle during isometric knee extension (SES=0.82) and Eccentric Knee Extension (SES=0.16). Liao et al. [46] examined 8 muscle strength parameters and 3 parameters for body functions and structures, with no significant results. Tankisheva et al. [50] reported better outcomes for the WBV than the control group: increase of isometric knee extension torque in paretic leg (week 6) (SES=1.74) and increase of Isokinetic knee extension strength (240°/s) in paretic leg (week 12) (SES=0.96), while in Van Nes et al. [49] both groups achieved similar improvements. This discrepancy is probably due to the difference in treatment duration and between the two control groups' treatments. In Van Nes et al. [49] we were not able to determine whether improvements are due to the conventional rehabilitation program (all participants took part in) or to additional WBV or to music therapy. Therefore we cannot say that WBV is a viable alternative to other types of therapy to deliver muscle strength improvements after stroke and other studies will be necessary to investigate the different effects varying WBV amplitude and duration.

### 4.3. Spasticity

In Chan et al. [44] the WBV significantly reduced spasticity measured with the Modified Ashworth Scale (MAS) (p≤.001) [54] and Visual Analogic Scale (VAS) (SES=1.96), The Hmax/Mmax ratio decreased significantly more in the WBV group in the unaffected leg only (SES=0.87), indicating a decrease in excitability of the stretch reflex pathway (Table 3). Participants were not “blind” to the treatment, so the increase of VAS can be a placebo effect. Of the 3 studies that measured spasticity after multiple WBV sessions [42, 43, 46, 50], only Pang et al. [42] reported beneficial effects on knee spasticity, but no effects on ankle spasticity evaluated with MAS. Liao et al. [46] applied the Kruskal-Wallis-Test to knee and ankle MAS ordinal data, providing an interquartile range for these parameters and showing no significant difference between the three groups examined.

Literature shows that because of its ordinal nature and because it is related to muscular activity and resistance in response to passive movements [55, 56], the MAS is probably not the best assessment for spasticity. To our knowledge this scale is the most used in selected studies, even if its results depend on the experience of the clinicians.

Evidences about the effects of WBV in reducing spasticity after stroke are insufficient in our review and it is impossible to declare the superiority of WBV compared to other rehabilitative processes.

### 4.4. Postural Control and Risk of Falls

Chan et al. [44] reported beneficial effects of a single session of WBV on postural control; however this was assessed by only measuring the distribution of weight between the legs (increase of total body weight percentage on affected side, SES=0.87, and decrease on unaffected side, SES=0.87) disregarding other important parameters such as biomechanical constraints: sensory orientation, walking balance, etc. We cannot exclude a placebo effect, since the participants were not “blind" to the intervention. The effects of multiple WBV sessions on balance are insufficient. None of the 5 studies [43, 45, 46, 49, 50] that measured balance outcomes showed significant differences between the groups after a treatment period of 6-12 weeks, suggesting that WBV does not provide poststroke improvements in postural control. Brogårdh et al. [43] and Marin et al. [45] used the Berg-Balance-Scale (BBS) as the main balance outcome. In these studies the level of disability at baseline was quite moderate, probably due to the inclusion criteria (Table 2), reducing the significance of the improvements.

In Liao et al. [46] the balance performance in daily activities was measured by the Mini- Balance-Evaluation-System-Test [57], producing nonsignificant results about WBV effects. However, the data demonstrated a decisive time-effect on increased balance levels (P <.001) for all groups. Van Nes et al. [49] showed that postural control improvements produced by WBV were similar to other types of physical activities. Tankisheva et al. [50] asserted a superiority of WBV compared to usual physical activity for improving balance in an upright posture using a swaying platform (SES=1.47). However, the authors did not explain why they only reported this improvement without dismissing other balance outcomes. Only Choi et al. [47] analyzed balance control in the sitting position, reporting significant improvements in the Modified-Reaching-Functional-Test (MFRT) after WBV: Anterior reach (SES=0.51); Nonparetic reach (SES=0.60); Paretic reach (SES=0.38). Only one study [41] measured the incidence of falls and reported negative results.

This was probably due to the fact that only 10% of the participants had at least one fall during the three months before treatment and the lack of any significant changes in motoneuron outcome variables.

The study of Lee G. [5], not considered by our systematic review because of an inadequate PEDro score (5/10), reported a significant increase in the equilibrium level measured with the Berg-Balance-Scale compared to pretreatment evaluations and the control group (difference of BBS score between baseline and follow-up: -6.00 ± 5.17 in the WBV group versus -0.56 ± 0.88 in the control group). We report this data because the research was conducted employing a platform that produced horizontal oscillations.

On the basis of these studies, we cannot recommend WBV to reduce the risk of falls in poststroke patients. (Lau et al. [41] reported a nonsignificant improvement in the incidence of falls during the period of follow-up between the WBV group and the control group who performed the same exercises, but without WBV.)

### 4.5. Functional Mobility

Only Chan et al. [44] investigated changes in functional mobility however, there were profound differences among groups before treatment with the WBV group having a greater level of disability than the control group (longer Timed “Up&Go” Test (TUG) and 10-Meter-Walk-Test (10MWT) times). The initial differences between groups may have influenced the outcome results, decrease of TUG (SES=1.80) and increase of 10MWT (maximal speed) (SES=0.79), since there may have been more room for improvement in individuals with more severe mobility limitations. Three studies [42, 43, 46] produced outcomes related to mobility, indicating that WBV does not confer advantages in this regard. This may be due to the fact that the exercises involved only part of functional components associated with gait, given the limitations of the vibratory devices. One study [48], combining WBV with Treadmill training, measured improvements by GAITRite (CIR systems Inc., USA, 2008) in Walking speed (SES=0.241), Step length of affected side (SES=0.337), and Stride length (SES=0.318). Although these results were positive, they need to be supported by other studies with larger sample sizes. Based on the available evidences it is not possible to draw positive conclusions regarding the effects of WBV therapy to improve mobility poststroke.

### 4.6. Somatosensory Threshold

The study by Van Nes et al. [49] showed improvements of somatosensory threshold in both WBV and control groups. No significative differences between groups were found.

All participants did the conventional rehabilitation program; so it was not possible to determine if the improvement of somatosensory threshold was due to conventional program, the additional use of WBV, or the music therapy program.

### 4.7. Activity and Participation

The initial intention of the study was to explore the literature based on the ICF Stroke Brief. Unfortunately, it was very challenging and very little information was available about the activity and participation (especially d599 self-care, d729 general interpersonal interaction, or d230 carrying out daily routine) in selected studies. However, the effects of WBV on participation in social activities were investigated by Brogårdh et al. [43] with negligible differences in scores of the Stroke-Impact-Scale between groups. Van Nes et al. [49], comparing WBV and music therapy, reported nonsignificant differences in the assessments of functional mobility and daily activities. Liao et al. [46] investigated several outcomes, but without any reported improvements; therefore it can be concluded that WBV therapy does not improve participation in social life for people with stroke.

### 4.8. Limitations

This systematic review included studies involving participants with nonhomogeneous characteristics, since studies with individuals with chronic and postacute stroke (disability level at baseline higher for the latter) were incorporated. Only 3 studies provided physiological explanations of the intervention protocol [41, 42, 46, 49]. Two studies [40, 50] had very low numbers of participants (≤20) reducing statistical power. In one study [44] there are profound differences between groups in impairment at the baseline. In other studies [42, 43, 46] there were detectable inadequacies in the protocols and instruments leading to poor correlations between Interventions and Outcomes. Some outcomes were described by ordinal variables, for which no data were provided on statistically significant improvements, only allowing a simple descriptive analysis.

## 5. Conclusions

By comparing WBV groups performing exercises during single or multiple sessions (4-12 weeks of treatment) to poststroke patients after the same exercises without WBV or other types of rehabilitation treatment, we are unable to demonstrate any significant systematic benefits from WBV treatment. This was mainly due to the heterogeneity of the studies completed to date. Though treatment with WBV appears safe and feasible and favourable in several outcomes, to our knowledge there are no sufficient evidences to support the integration of WBV in poststroke rehabilitation programs.

We are not able to highlight the differences between a synchronous and asynchronous vibration treatment, because there were no studies designed to investigate this aspect. Future RCTs may consider this topic and also the other parameters of the vibration platform, by continuing the research started by Liao 2016 [44] who investigated the effects of different stimulus intensities.

Future studies need to use outcome measures with good psychometric properties such as multiple measures for the same outcome, a statistically useful number of participants, and homogeneous disability characteristics for participants.

## Figures and Tables

**Table 1:** Results of the quality assessment of the included studies.

Criterion	**Study**									
	**Comparison 1 (7 studies)**								**Comparison 2 (2 studies)**	
	The Control Group make exercise under the same condition as the Whole-Body Vibration Group, but without Whole-Body Vibration or with sham vibration								The Control Group make other forms of exercise or physical activity	
	**Tihanyi et al, 2007 **[40]	**Lau et al, 2012** [41] **and Pang et al, 2013** [42]	**Brogårdh et al, 2012 **[43]	**Chan et al, 2012 **[44]	**Marín et al, 2013 **[45]	**Liao et al, 2016 **[46]	**Choi et al, 2014 **[47]	**Choi W et al, 2017 **[48]	**Van Nes et al, 2006 **[49]	**Tankisheva et al, 2014** [50]
eligibility criteria (it does not contribute to total PEDro score)	x	x	x	x	x	x	x	x	x	x
random allocation	x	x	x	x	x	x	x	x	x	x
concealed allocation	x	x	x	x	x	x		x	x	x
baseline comparability	x	x		x	x	x	x	x	x	x
subjects blinding			x							
therapist blinding			x	x						
assessor blinding		x	x	x	x	x		x	x	x
adequate follow-up (>85%)	x	x	x		x	x	x	x	x	x
intention-to-treat analysis		x	x	x	x	x	x	x	x	
between-group comparisons	x	x	x	x	x	x	x	x	x	x
points estimates and measure of variability provided	x	x	x	x	x	x	x	x	x	x
total PEDro score	6/10	8/10	9/10	8/10	8/10	8/10	6/10	8/10	8/10	7/10
sample size ≥ 50	No	Yes	No	No	No	Yes	No	No	Yes	No

**Table 2:** Characteristics of participants in the reviewed studies and summary of immediate effects of a single/multiple session/s of WBV in people with stroke  ^I^.

**Study, Type of study, and Recruitment**	**Participant Characteristic** **s** ^**II**^			**Inclusion Criteria**	**Exclusion Criteria**	**Severity of impairments at Baselin** **e** ^**II**^		**Outcome Measures** ^**IV**^		**Conclusion**
	**Sample size and groups**	**Age (years)**	**Poststroke duration**			**Measure**	**Values**	**No Significant Findings**	**Significant Findings**	
**Studies that assessed the effects of a single WBV session (comparison 1)**										

Tihanyi et al, 2007 [40] ^**VII**^ RCT Rehabilitation Center	16 Subacute stroke (10 men, 6 women) WBV 8 Control 8	58.2 ± 9.4	Postacute stroke (days) 27.2 ± 10.4	First-time Stroke; 14-50 days after stroke onset; FIM score at admission of 60-110	Unstable cardiac conditions; Peripheral arterial disease; Severe dementia; Unable to follow simple commands; Painful orthopedic conditions involving the pelvis and lower limbs	BI (0-100)^III^ = 46 (25-85)^III^ FIM score (18-126)^III^	46 (25-85)^III^ 84 (63-110)^III^	Mechanical work during eccentric contraction	↑Maximum isometric knee extension torque (SES=0.50)^IV^; ↑Maximum eccentric knee extension torque (SES=0.46); ↑Rate of torque development (SES=0.08); ↑Maximum voluntary eccentric torque at 60° of knee flexion (SES=0.46); ↓Coactivation quotient of BF during: isometric knee extension (SES=0.82) Eccentric Knee Extension (SES=0.16)	“A single bout of WBV can transiently increase voluntary force and muscle activation on the quadriceps muscle affected by a stroke"

Chan et al, 2012 [44] RCT Rehabilitation Center	30 Chronic Stroke (21 men, 9 women) WBV 15 Control 15	WBV 56.07 ± 11.04 Control 54.93 ± 7.45	Chronic stroke (months) WBV 30.40 ± 25.80 Control 38.87 ± 38.22	First stroke; Stroke onset >6 months previously; Ankle MAS score ≥2; Able to ambulate with or without assistive device for at least 100m; MMSE score ≥24; No joint contractures; Able to complete functional walking tests	Gallbladder or kidney stones; Recent leg fractures; Internal fixation implants; Cardiac pacemaker, intractable hypertension; Recent thromboembolism; Recent infectious diseases	Ambulatory device use (n) Regular cane Quad cane MAS score (0-5)	6 8 2.4 ± 0.5	GS H-reflex in both legs; GS Hmax/Mmax ratio on affected side; Achilles deep tendon reflex on affected side; Cadence	↓GS Hmax/Mmax ratio on unaffected side (SES=0.87)^IV^; ↓MAS^V^; ↓VAS (perceived spasticity) (SES=1.96); ↓Time to complete TUG (SES=1.80); ↑10MWT (maximal speed) (SES=0.79); ↑TBW% on affected side (SES=0.87); ↓TBW% on unaffected side (SES=0.87)	“A single session of WBV can reduce ankle plantar- flexion spasticity in chronic stroke patients, thereby potentially increasing ambulatory capacity"

**Studies that assessed the effects of multiple WBV sessions (comparison 1)**										

Lau et al, 2012 [41] and Pang et al, 2013 [42] Single-Blinded RCT Local Stroke Self- Help Group	82 Chronic Stroke (58 men, 24 women) WBV41 Control 41	WBV 57.3 ± 11.3 Control 57.4 ± 11.1	Chronic stroke (months) WBV 4.6 ± 3.5 Control 5.3 ± 4.2	Hemispheric stroke; Stroke onset >6 months previously; Medically stable; AMT score ≥6; Age ≥18 years; Able to stand independently with or without aids for at least 90sec	Other neurological conditions; Serious musculoskeletal conditions; Pain that affected the performance of physical activities; Metal implants or recent fractures in the lower extremity; Vestibular disorders; Peripheral vascular disease; Other serious illness; Pregnancy	Walking aids indoors (none/cane/quad cane) CMSA leg score (out of 7) CMSA foot score (out of 7) participants with at least 1 fall in prev. 3 months (n) FAC score (1–5) BBS score (0–56) Knee concentric extension peak power (W/kg): Paretic leg Nonparetic leg	65/8/9 4 (3–6)^III^ 3 (1–6)^III^ 4 5 (3–5)^III^ 50.8 ± 6.7 0.65 ± 0.33 1.18 ± 0.45	BBS; Limit of stability test (MVL, EPE, MXE, DCL); 6MWT; 10MWT (comfortable speed); CMSA on paretic leg and foot; Ankle spasticity (MAS); ABC; CTx; BAP; Paretic leg isometric muscle strength (Knee extension, Knee flexion, Paretic and non-paretic knee peak power, Concentric extension, Concentric flexion, Eccentric extension, Eccentric flexion); Incidence of falls	↓ Knee MAS ^V^ (week 12)	The addition of WBV to a leg exercise protocol was no more effective in improving neuromotor performance, bone turnover, and paretic leg motor function and reducing the incidence of falls than leg exercises alone in patients with chronic stroke who have mild to moderate motor impairments. WBV may have potential to modulate spasticity.

Brogårdh et al, 2012 [43] Double-Blinded RCT Rehabilitation Center	31 Chronic Stroke (25 men, 6 women) WBV 16 Control 15	WBV 61.3 ± 8.5 Control 63.9 ± 5.8	Chronic stroke (months) WBV 37.4 ± 31.8 Control 33.1 ± 29.2	Able to walk ≥300m; ≥10% self- perceived muscle weakness in the knee extensors or knee flexors in the paretic leg; Not engaging in any heavy resistance or high- intensity training;	Epilepsy; Cardiac disease; Cardiac pacemaker; Osteoarthritis in the lower limbs; Knee or hip joint replacement; Thrombosis in the lower limbs in previous 6 months	FIM score (18–126) BBS score (0–56) Isometric knee extension (Nm): Paretic leg Nonparetic leg	83.3 ± 3.2 51.2 ± 2.3 98.2 ± 33.7 144.8 ± 36.2	MAS; BBS; Muscle strenght (Isokinetic knee extension in both legs(60°/s), Isokinetic knee flexion in both legs (60°/s), maximum isokinetic knee extension in both legs); TUG; 10MWT (comfortable and maximal speed); 6MWT; SIS		Six weeks of WBV training had small treatment affects on balance and gait performance in individuals with chronic stroke but was not more effective than a placebo vibrating platform

Marín et al, 2013 [45] RCT Rehabilitation Center	20 Chronic Stroke (11 men, 9 women) WBV11 Control 9	WBV 62.4 ± 10.7 Control 64.4 ± 7.6	Chronic stroke (years) WBV 4.3 ± 2.0 Control 4.3 ± 3.0	Stroke onset ≥6 months previously; NIHSS score > 1 and <20	Dementia or severe cognitive impairment; Knee joint pain; Unable to remain standing without external support for ≥30 s	NIHSS score (0–42) BBS score (0–56)	1.3 ± 0.5 46.1 ± 9.1	Muscle thickness of RF, VL, and MG in both legs; Maximum isokinetic knee extension strength; BBS		“WBV exercise did not augment the increase in neuromuscular performance and lower limb muscle architecture induced by isometric exercise alone in stroke patients."

Choi et al, 2014 [47] RCT Rehabilitation Hospital	30 Chronic Stroke (16 men, 14 women) WBV 15 Control 15	WBV 62.8 ± 9.0 Control 65.1 ± 15.7	Chronic stroke (months) WBV 13.0 ± 5.4 Control 12.6 ± 5.7	Stroke onset >6 months previously; ability to sit independently for at least 10 minutes; no participation in any balance training program during the previous six months; no orthopedic problems, such as a fracture, deformity, or severe osteoarthritis; Korean version of MMSE score ≥21	Comorbidity or disability other than stroke; Uncontrolled health condition for which vibration is contraindicated	Static Sitting Balance - COP: Velocity average (cm/s) Total Path Lenght (cm) Dynamic sitting balance MFRT (cm): MFRT-A MFRT-N MFRT-P	3.0 ± 0.3 89.4 ± 11.5 23.5 ± 15.0 12.2 ± 7.3 10.3 ± 7.1	COP sway average velocity; COP sway path length	↑ MFRT Anterior reach (SES=0.51); ↑ MFRT Non-paretic reach (SES=0.60); ↑ MFRT Paretic reach (SES=0.38)	Four weeks of task oriented training with WBV had no significant effects on static sitting balance. WBV improved reach task
Liao, 2016 [46] Single-Blinded RCT Stroke Association	84 Chronic Stroke (62 men, 22 women) LWBV 28 HWBV 28 Control 28	LWBV 60.8 ± 8.3 HWBV 62.9 ± 10.2 Control 59.8 ± 9.1	Chronic stroke (years) LWBV 8.5 ± 5.2 HWBV 8.1 ± 4.2 Control 9.0 ± 4.6	Hemispheric stroke onset >6 months previously; Age ≥18 years; Community dweller; AMT score ≥ 6; Able to stand independently with or without aids for at least 90 sec	Brainstem or cerebellar stroke; Other neurological disorders; Neoplasms; Pregnancy; Pain that affected the ability to participate in physical activities; Vestibular conditions; Metal implants or recent fractures in the lower limbs; Other serious medical problems; Severe cardiovascular diseases (pacemaker and uncontrolled hypertension)	CMSA lower limb score (2–14) Paretic knee MAS of spasticity (0–4): 0/1/1.5/2/3/4 (n) Median (IQR) Paretic ankle MAS of spasticity (0–4): 0/1/1.5/2/3/4 (n) Median (IQR) Functional ambulation category (0–5) Walking aids (none/cane/quad/frame/ rollators/wheelchair) (n) participants with at least 1 fall in prev 12 months (n) Total number of comorbid medical conditions Hypertension (n) High cholesterol (n) Total number of medications Antihypertensive agents (n) Hypolipidemic agents (n) Antidiabetic agents (n) Muscle relaxants (n)	9 (7–11.75)^III^ 24/28/23/9/0/0 1 (0–2) 7/10/35/24/7/1 2 (2–2) 5 (4–5) indoor 75/6/3/0/0/0 outdoor 31/38/3/6/0/6 30 1.8 ± 1.0 16 50 4.3 ± 1.8 31 50 14 6	Paretic and Non-paretic leg muscle strength (Isometric extension at 70°, Isometric flexion at 70°, Isometric extension at 30°, Isometric flexion at 30°, Concentric extension, Concentric flexion, Eccentric extension, Eccentric flexion); Body functions and structures (Knee spasticity median (IQR), Ankle spasticity median (IQR), VO_2_ during 6MWT); Activity (TUG, 6MWT distance, Mini-BESTest); Participation (ABC, FAI, CHIEF-C, SF-12, PCS, MCS)		“The addition of the 30- session WBV paradigm to the leg exercise protocol was no more effective in enhancing body functions/structures, activity, and participation than leg exercises alone in chronic stroke patients with mild to moderate motor impairments.”
Choi W et al, 2017 [48] RCT not specified	30 Chronic (19 men, 11 women) WBV 15 Control 16	WBV 51.93 ± 8.35 Control 53.67 ± 7.38	Chronic stroke (months) WBV 25.13 ± 9.25 Control 22.53 ± 10.27	Gait deviation; stroke onset >6 months previously; ability to walk more than 30 seconds at >0.8 km/h; ability to understand the nature of the intervention and perform the protocol independently; MMSE score ≥21	participation in similar experiments during the previous 6 months; fracture, infectious disease, cardiac pacemakers, vestibular disorders, cerebellar diseases, visual and auditory problems, walking disability due to orthopedic problems, chronic pain, contracture in the lower extremity joints.	Walking speed (cm/s) Cadence (step/min) Step length affected side (cm) Step length less affected side (cm) Stride length (cm) Single limb support affected side (%) Single limb support less affected side (%) double limbs support (%)	51.62 ± 25.61 84.33 ± 23.01 35.50 ± 13.05 35.47 ± 12.46 71.22 ± 24.02 23.02 ± 7.46 33.28 ± 8.03 43.38 ± 14.16	6MWT	↑ GAITRite: Walking speed (SES=0.241), Step length of affected side (SES=0.337), Stride length (SES=0.318)	6 weeks of WBV combined with treadmill training might be a more intensive and effective training program than treadmill training to improve the walking performance of patience with chronic stroke.

**Studies that assessed the effects of multiple WBV sessions (comparison 2)**										

Van Nes et al, 2006 [49] RCT Rehabilitation	53 Acute Stroke (30 men, 23 women) WBV 27 ETM 26	WBV 59.7 ± 12.3 ETM 62.6 ± 7.6	Post-acute stroke (days) 36.6 ± 9.7	Stroke onset <6 weeks previously; Moderate or severe balance impairments (BBS score <40)	Gallbladder or kidney stones; Unable to follow simple verbal instructions; Cardiac pacemaker; Non-Stroke-related sensory or motor impairments; Medication that could interfere with postural control; Malignancies; Pregnancy; Recent fractures	MI (0-100) MAS (0-5) Knee Flexion MAS (0-5) Knee extension MAS (0-5) Ankle DF MAS (0-5) Ankle PF BBS score (0-56) BI (0-20) Trunk Control Test (0-100) RMI score (0-15) FAC score (0-5)	49.0 ±28.6 0 (0-3)^III^ 0 (0-4)^III^ 1 (0-4)^III^ 0 (0-2)^III^ 23.8 ±16.8 10.1 ± 3.4 72.3 ± 25.0 5.3 ± 3.1 1 (0-4)^III^	BBS, BI; Rivermead Mobility Index; Trunk Control Test; FAC; Motricity Index; Somatosensory threshold of affected leg		WBV was “not more effective in enhancing recovery of balance and activities of daily living than the same amount of exercise therapy on music in the postacute phase of stroke."
Tankisheva et al, 2014 [50] RCT Rehabilitation Center	15 Chronic (10 men, 5 women) WBV 7 Control 9	WBV 57.4 ± 13.0 Control 65.3 ± 3.7	Chronic stroke (months) WBV 7.7 ± 8.6 Control 5.2 ± 3.6	Aged 40-75 years; First-ever stroke; Stroke onset >6 months previously; Medically stable; Able to stand independently with or without aids for at least 20 min; Able to perform the experimental treatment independently	Cardiac pacemaker; Acute hernia; Diabetes; Tumors; Acute thrombotic diseases; Severe heart and vascular diseases; Other neurologic disorders (rheumatoid arthritis, arthrosis); Osteoarthritis; Discopathy; Spondylosis	Isometric knee extension (Nm): Paretic leg Nonparetic leg BI (0-20) FAC score (1-6) Brunnström-Fugl-Meyer test score Ashworth scale composite score (0- 24) SOT score: C1 C2 C3 C4 C5 C6	96.4 ± 19.6 135.7 ± 16.0 90.4 ± 10.2 5 (3-5)^III^ 22.9 ± 5.3 4.5 (0-14)^III^ 92.7 ± 2.4 89.9 ± 3.0 89.4 ± 4.1 73.8 ± 6.5 41.8 ± 28.9 51.3 ± 19.5	MAS; Muscle strength; Isokinetic knee extension in both legs (60°/s); Isokinetic knee flexion in both legs (60°/s); Isokinetic knee extension in nonparetic leg; Isokinetic knee flexion in both legs; Isokinetic knee extension in nonparetic leg (240°/s); Isokinetic knee flexion in both legs (240°/s); SOT; Equilibrium scores (%) in conditions 1,2,3,5 and 6	↑ Isometric knee extension torque in paretic leg (week 6) (SES=1.74); ↑ Isokinetic knee extension strength (240°/s) in paretic leg (week 12) (SES=0.96); ↑ Equilibrium scores (%) in condition 4: normal vision and sway- referenced support surface (week 6) (SES=1.47)^VI^	Six weeks of intensive WBV might “potentially be a safe and feasible way to increase some aspect of lower limb muscle strength and postural control in adults with chronic stroke”

^I^  6MWT: Six-Minute walk Test, 10MWT: 10-Meter Walk Test, ABC: Activities-Specific Balance Confidence Scale, AMT: Abbreviated Mental Test, BAP: Bone-specific Alkaline Phosphatase, BBS: Berg Balance Scale, BF: Biceps Femoris Muscle, BI: Barthel Index, C: Condition, CGS: Comfortable Gait Speed, CHIEF-C: Chinese version of the Craig Hospital Inventory of Environmental Factors, CMSA: Chedoke-McMaster Stroke Assessment, COP: Center Of Pressure, CTx: Serum C-Telopeptide of type I collagen cross-links, DCL: Directional Control, EPE: Endpoint Excursion, F: female, M: male, FAI: Frenchay Activity Index, FGS: Fast Gait Speed, FAC: Functional Ambulation Categories, FIM: Functional independence Measure, GS: Gastrocnemius-soleus Muscle, H-reflex: Hoffmann reflex, Hmax/Mmax ratio: maximum H-reflex/maximum M-response ratio, IQR: Interquartile range, L: left, R: right, MAS: Modified Ashworth Scale, MCS: Mental Health Composite Score, MFRT: Modified Functional Reach Test, MFRT-A: MFRT-Anterior Reach, MFRT-N: MFRT-Nonparetic Reach, MFRT-P: MFRT-Paretic Reach, MG: Medial Gastrocnemius Muscle, MI: Motricity Index, Mini-BESTest: Mini Balance Evaluation System Test, MMSE: Mini-Mental State Examination, MVL: Movement Velocity, MXE: Maximum Excursion, NIHSS: National Institutes of Health Stroke Scale, NP/P: Nonparetic to Paretic, NR: not reported, PCS: Physical Composite Score, RF: Rectus Femoris Muscle, RMI: Rivermead Mobility Index, SES: Standardized Effect Size, SF-12: Short-Form 12 Health Survey, version 2 (Chinese version), SIS: Stroke Impact Scale, SOT: Sensory Organization Test, TUG: Timed “Up & Go” Test, TBW%: Percentage of total body weight, VAS: Visual Analog Scale, VL: Vastus Lateralis Muscle, WBV: Whole Body Vibration Group, LWBW: Low-Intensity Whole Body Vibration, HWBW: High-Intensity Whole Body Vibration, ↓: increase, ↑: decrease.

^II^  Mean±SD presented unless indicated otherwise.

^III^  Median (range).

^IV^  The results shown in this table refer to the difference between the WBV and comparison groups. The SES was calculated from the mean and standard deviation of the change scores unless indicated otherwise.

^V^  The SES was not reported because MAS is an ordinal variable.

^VI^  The SES for this particular outcome was reported in the text by the authors.

^VII^  The electromyographic amplitude data of individual muscles were not included because they were not normalized, making it difficult to compare groups.

**Table 3:** Training protocol for WBV protocol and comparison group^I^.

**Study**	**Protocol for WBV group**								**Protocol for Comparison Group**
	**WBV Treatment**						**Additional Treatment**	**Supervision**	
	**Frequency of Sessions × Duration of Program**	**No. of Vibration Bouts × Duration per Bout**	**Rest**	**Frequency (Hz), Amplitude (mm), and Peak Acceleration (g) of Vibration Signals**	**WBV type and commercial name**	**Posture**			
**Studies that assessed the effects of a single WBV session (comparison 1)**									

Tihanyi et al, 2007 [40]	Single session	6 bouts × 1min	120s	20Hz 2.5mm 4.0*g*	Synchronous Vertical Nemes-Bosco	Standing on the platform with knees slightly flexed at 40° and shifting body mass to the paretic leg	None	NR	Same exercise but without vibration

Chan et al, 2012 [44]	Single session	2 bouts × 10min	60s	12Hz 4mm 2.3*g*	Synchronous Vertical AV 001 - Body Green	Positioned on the platform in a semi-squatting position with buttock support and were kept in an upright position with even weight distribution on both feet	None	NR	Followed the same procedures, but the vibration machine was not turned on

**Studies that assessed the effects of multiple WBV sessions (comparison 1)**									

Lau et al, 2012 [41] and Pang et al, 2013 [42]	3/week × 8weeks	6bouts × 1.5min to 6bouts × 2.5min	3 - 4.5 Min	20 - 30Hz 0.44 - 0.60mm 1.0 - 1.6*g*	Synchronous Vertical Jet Vibe System	Side-to-side weight shift, semi- squat, forward and backward, weight shift, forward lunge, standing on one leg, deep squat	1.5min of warm-up exercises (general mobilization and stretching) in a sitting position	Therapist	Performed the same exercises on the same WBV platform as the WBV group but without vibration

Brogårdh et al, 2012 [43]	2/week × 6 weeks	4 bouts × 40s to 12 bouts × 60s	60s	25Hz 3.75mm 9.2*g*	Synchronous Vertical Xrsize	Standing barefoot on the platforms in a static position with the knees flexed 45°-60° and with handhold support, if needed	None	Physical Therapist	Some exercises on a vibration platform with an amplitude of 0.20mm and a frequency of 25Hz

Marín et al, 2013 [45]	1/week (from week 1 to week 7) 2/week (from week 8 to week 12)	sessions 1-2: 4 bouts × 30s sessions 3-4: 5 bouts × 30s sessions 5-6: 5 bouts × 50s sessions 7-8: 5 bouts × 60s sessions 9-12: 6 bouts × 60s sessions 13-17: 7 bouts × 60s	60s	5 - 21Hz 2 - 3mm 0.2 - 5.3*g*	Side-alternating Vertical Galileo Home	Standing on a vibration platform with knee flexion of 30°	10 × 2 hours rehabilitation sessions per month	Therapist	Performed the same exercises as that of WBV group, but was not exposed to vibration, + 10 × 2 hours rehabilitation sessions per month

Choi et al, 2014 [47]	5/week × 4 weeks	4 bouts × 3min	60s	15 - 22Hz 0 - 5.8mm (*g*) Peak: NR	Side-alternating Vertical Galileo Pro	Sitting alone at a table and correcting body alignment, reaching task beyond arm's length using the non-paretic side, same reaching task using the paretic side, bilateral reaching task	None	Researcher	Performed the same exercises as that of WBV group, but was not exposed to vibration

Liao, 2016 [46]	3/week × 10 weeks	sessions 1-15: 8 bouts × 90s sessions 16-30: 12 bouts × 90s	90s	LWBV Group: 20Hz, 1mm, 1.61*g* HWBV Group: 30Hz, 1mm, 3.62*g*	Synchronous Vertical Gymna Fitvibe Medical System	Dynamic weight shift side to side, Dynamic deep squat, Dynamic forward and backward weight shift, Static semisquat (starting position: standing on the WBV platform with feet placed width apart at shoulder width, with bilateral knees flexed at 10°)	10min of warm-up + 10min of cool-down exercises (general stretching exercises in a sitting position and exercises using a cycle ergometer)	Researcher	Performed the same exercises on the same WBV platform as the WBV group but without vibration

Choi W et al, 2017 [48]	3/week x 6 weeks	6 bouts x 45s	60s	session 1-2: 20Hz, 3mm session 3-4: 25Hz, 3mm session 5-6: 30Hz, 3mm (*g*) Peak: NR	Side-alternating Galileo 2000	Weight shift side to side, Squat (knee joint 45°flexion), Forward and backward weight shift, Forward lunges, One-leg standing (alternately), Deep squat (knee joint 90°flexion)	15min of warm-up + 20min of Treadmill Training	Physical Therapist	Performed the same exercises as that of WBV group, but was not exposed to vibration

**Studies that assessed the effects of multiple WBV sessions (comparison 2)**									

Van Nes et al, 2006 [49]	5/week × 6 weeks	4 bouts × 45s	60s	30Hz 3mm 10.9*g*	Side-alternating Vertical Galileo 900	Standing on the platform with the knees slightly flexed	None	Physical Therapist	Exercise therapy with music: regular exercises for the trunk, arm, and leg muscles

Tankisheva et al, 2014 [50]	3/week × 6 weeks	sessions 1-12: 5 bouts × 30s sessions 13-18: 17 bouts × 60s	NR	sessions 1-12: 35Hz, 1.7mm, 8.4*g* sessions 13-18: 40Hz, 2.5mm, 16.1*g*	Synchronous Vertical Power Plate	Standing on their toes, knee flexion of 50°-60° (high squat), knee flexion of 90° (deep squat), wide-stance squat, and 1-legged squat	None	Trainer	Participants in the control group were not involved in any additional training program and were asked not to change their lifestyle

^I^Mean±SD presented unless indicated otherwise. *g*: 1 unit of gravitational constant (9.8 m/s^2^), NR: not reported, WBV: Whole Body Vibration, LWBW: Low-Intensity Whole Body Vibration, HWBW; High-Intensity Whole Body Vibration.

## Supplementary Materials

Supplementary Materials (i) PRISMA Checklists: this file is a brief description of 27 PRISMA items pertaining to the content of a systematic review, indicating where it can be found in the manuscript (pages, tables, and figure), provided as requested by Preferred Reporting Items for Systematic Reviews and Meta-Analyses (PRISMA) Statement. (ii) PubMed Search String: this file is the search string used for the initial research in the first database, provided as requested by revisers. (iii) PubMed Screenshot File 2018-05-12 at 14.54.13: this file is a picture presenting the PubMed search results obtained by applying the declared filters with the PubMed String Search, on 2018-05-12 at 14.54.13. (iv) PubMed Result.csv: this file is a text file-based file format used for importing and exporting of our PubMed search results.
