# Supplementary material for: Effects of Single or Multiple Sessions of Whole Body Vibration in Stroke: Is There Any Evidence to Support the Clinical Use in Rehabilitation?
Source: Rehabil Res Pract. 2018 Jul 30;2018:8491859. doi: 10.1155/2018/8491859 (PMC6091286; doi:10.1155/2018/8491859)
Supplement: Supplementary Materials — (i) PRISMA Checklists: this file is a brief description of 27 PRISMA items pertaining to the content of a systematic review, indicating where it can be found in the manuscript (pages, tables, and figure), provided as requested by Preferred Reporting Items for Systematic Reviews and Meta-Analyses (PRISMA) Statement. (ii) PubMed Search String: this file is the search string used for the initial research in the first database, provided as requested by revisers. (iii) PubMed Screenshot File 2018-05-12 at 14.54.13: this file is a picture presenting the PubMed search results obtained by applying the declared filters with the PubMed String Search, on 2018-05-12 at 14.54.13. (iv) PubMed Result.csv: this file is a text file-based file format used for importing and exporting of our PubMed search results. [file 8491859.f1.zip › Supplementary Material/PubMed Search String.docx]

**PubMed Search String**

(("nervous system diseases"[MeSH Terms] OR ("nervous"[All Fields] AND "system"[All Fields] AND "diseases"[All Fields]) OR "nervous system diseases"[All Fields] OR ("nervous"[All Fields] AND "system"[All Fields] AND "disease"[All Fields]) OR "nervous system disease"[All Fields]) OR ("stroke"[MeSH Terms] OR "stroke"[All Fields])) AND ((whole[All Fields] AND ("human body"[MeSH Terms] OR ("human"[All Fields] AND "body"[All Fields]) OR "human body"[All Fields] OR "body"[All Fields]) AND ("vibration"[MeSH Terms] OR "vibration"[All Fields])) OR ("vibration"[MeSH Terms] OR "vibration"[All Fields]) OR (("vibration"[MeSH Terms] OR "vibration"[All Fields]) AND platform[All Fields])) AND (("placebos"[MeSH Terms] OR "placebos"[All Fields] OR ("sham"[All Fields] AND "therapy"[All Fields]) OR "sham therapy"[All Fields]) OR ("rehabilitation"[MeSH Terms] OR "rehabilitation"[All Fields] OR ("rehabilitation"[All Fields] AND "therapy"[All Fields]) OR "rehabilitation therapy"[All Fields])) AND (("gait"[MeSH Terms] OR "gait"[All Fields]) OR ("Balance"[Journal] OR "balance"[All Fields]) OR (("muscles"[MeSH Terms] OR "muscles"[All Fields] OR "muscle"[All Fields]) AND performance[All Fields]) OR ("muscle spasticity"[MeSH Terms] OR ("muscle"[All Fields] AND "spasticity"[All Fields]) OR "muscle spasticity"[All Fields] OR "spasticity"[All Fields]) OR ("bone remodeling"[MeSH Terms] OR ("bone"[All Fields] AND "remodeling"[All Fields]) OR "bone remodeling"[All Fields] OR ("bone"[All Fields] AND "turnover"[All Fields]) OR "bone turnover"[All Fields]) OR (postural[All Fields] AND ("prevention and control"[Subheading] OR ("prevention"[All Fields] AND "control"[All Fields]) OR "prevention and control"[All Fields] OR "control"[All Fields] OR "control groups"[MeSH Terms] OR ("control"[All Fields] AND "groups"[All Fields]) OR "control groups"[All Fields])) OR ("muscle strength"[MeSH Terms] OR ("muscle"[All Fields] AND "strength"[All Fields]) OR "muscle strength"[All Fields]))
