# Supplementary figures and images for: Effects of Single or Multiple Sessions of Whole Body Vibration in Stroke: Is There Any Evidence to Support the Clinical Use in Rehabilitation?
Source: Rehabil Res Pract. 2018 Jul 30;2018:8491859. doi: 10.1155/2018/8491859 (PMC6091286; doi:10.1155/2018/8491859)

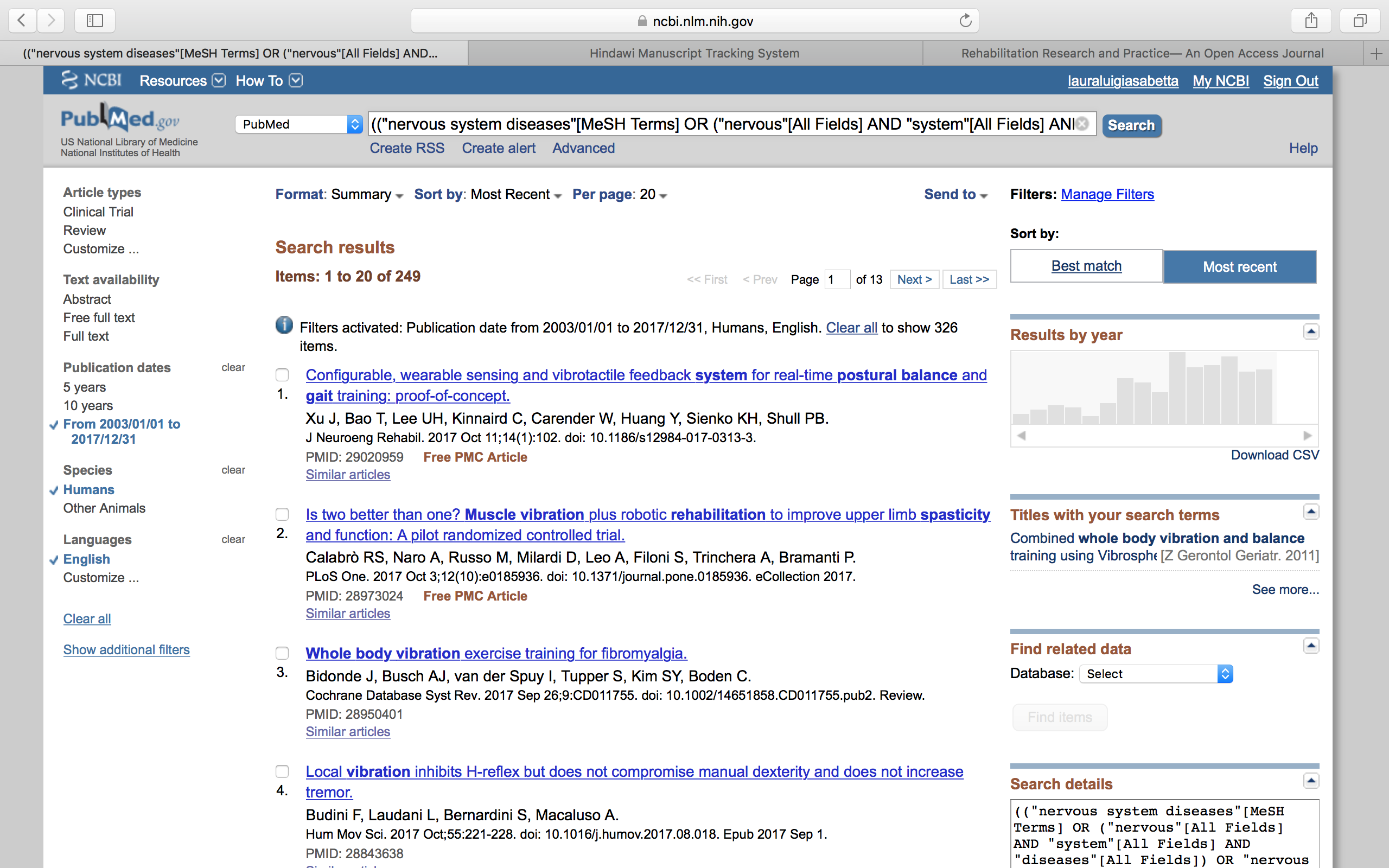

Supplement: Supplementary Materials — (i) PRISMA Checklists: this file is a brief description of 27 PRISMA items pertaining to the content of a systematic review, indicating where it can be found in the manuscript (pages, tables, and figure), provided as requested by Preferred Reporting Items for Systematic Reviews and Meta-Analyses (PRISMA) Statement. (ii) PubMed Search String: this file is the search string used for the initial research in the first database, provided as requested by revisers. (iii) PubMed Screenshot File 2018-05-12 at 14.54.13: this file is a picture presenting the PubMed search results obtained by applying the declared filters with the PubMed String Search, on 2018-05-12 at 14.54.13. (iv) PubMed Result.csv: this file is a text file-based file format used for importing and exporting of our PubMed search results. [file 8491859.f1.zip › Supplementary Material/PubMed Screenshot File 2018-05-12 at 14.54.13.png]
